# Yingyangbao Intervention Was Associated with the Improvement of Body Growth and Nutrition Status of Infants and Young Children in Poor Rural Areas of China: The Annual Comparison from 2012 to 2020

**DOI:** 10.3390/nu15102350

**Published:** 2023-05-17

**Authors:** Ou Wang, Jian Huang, Junsheng Huo, Di Chen, Yifan Xu, Jing Sun

**Affiliations:** NHC Key Laboratory of Trace Element Nutrition, National Institute for Nutrition and Health, Chinese Center for Disease Control and Prevention, Beijing 100050, China; wangou@ninh.chinacdc.cn (O.W.); huangjian@ninh.chinacdc.cn (J.H.); huojs@ninh.chinacdc.cn (J.H.); chendi@ninh.chinacdc.cn (D.C.); xuyf@ninh.chinacdc.cn (Y.X.)

**Keywords:** Yingyangbao, infants and young children, growth, malnutrition status

## Abstract

Yingyangbao (YYB) is a soy-based micronutrient-fortified powder used in the Nutrition Improvement Project on Children in Poor Areas of China. After the baseline study in 2012, YYB intervention gradually spread to 21 provinces in China. From 2015 to 2020, a secular trend study was carried out to evaluate the body growth and nutritional status of 6–23-month-old infants and young children (IYC) under YYB intervention. The aim of this research was to investigate whether YYB intervention was associated with the improvement of body growth and development in large populations from broad areas with national and multi-year survey results. The anthropometric data collected in the baseline study and cross-sectional surveys were compared, and the correlation between YYB intake amount and body growth were analyzed. Compared with the results of the baseline study, the 6–23-month-old IYC under YYB intervention showed a significant increase in body weight, body length and Z scores since 2015, and the stunting rate decreased from 9.7% in 2012 to 5.3% in 2020. Significantly positive correlations were observed between the YYB intake amount and the body growth indicators. Therefore, YYB intervention was associated with the improvement of body growth and nutrition status of Chinese IYC. In the future, long-term and continuous efforts are still needed to further reveal the health benefits of YYB in depth.

## 1. Introduction

Early life nutrition is one of the dominant factors that decide the body growth and development of infants and young children (IYC) and may shape overall health over the course of one’s life [1,2,3]. Micronutrient fortification is a consensus-based method to prevent or improve nutrient deficiency problems for children. Chinese IYC have been included in high malnutrition risk groups with high rates of anemia and stunting, especially in poor rural regions, in past decades. Yingyangbao (YYB) product is a soy-based powder with fortified vitamins and minerals [4,5]. One sachet of YYB weighs 12 g and contains 3.0 g protein, 7.5 mg iron, 200.0 mg calcium, 3.0 mg zinc, 250.0 µg RE vitamin A, 5.0 µg vitamin D, 75.0 µg folic acid, 0.5 mg Vitamin B_1_, 0.5 mg Vitamin B_2_ and 0.5 µg Vitamin B_12_. It was purposely designed for the IYC in poor rural regions based on their nutrition status and requirements.

Nutritionist Chen Chunming’s team from China’s CDC observed the intervention effect of a soy-based micronutrient-fortified powder on IYC in five poor counties in Gansu, a less economically developed province in west China, from 2001 to 2003. An energy-matched rice powder was used as the control group. Data from this carefully designed study showed that the hemoglobin level, anemia rate, Z score of height for age and cognitive development were all significantly improved by the micronutrient-fortified soybean powder compared to the control group [6,7,8]. After this study, the formula of the soy-based micronutrient powder was modified and named YYB by the team [9]. In 2008, the Wenchuan earthquake resulted in an anemia rate of up to 80% and other serious nutrition problems in eight counties from the provinces of Sichuan, Gansu and Shaanxi. Two months after the disaster, YYB was used as an emergency nutrition intervention complementary food by the health department of the government and was freely distributed to approximately 30,000 families with 6–23-month-old infants in those eight counties. The results from the earthquake-stricken areas indicated a remarkable nutrition improvement under YYB intervention [10,11]. In addition, YYB, as a high-tech product produced in large urban cities, was difficult to get to poor rural areas by market supply chains. In China, a so-called three-layer system has already been well-established among county public health departments, township health centers and village clinics. Therefore, the application of YYB intervention could be achieved highly efficiently through the three-layer system, which is responsible for YYB distribution, feeding knowledge communication and nutrition monitoring. The three-layer distribution could integrate YYB manufacturers and infants’ families and support YYB intervention feasibly and sustainably.

With the development of China, the nutritional status of Chinese children exhibited big improvement, but anemia and stunting were still nutrition problems among children under five years old. Therefore, based on these previous studies about YYB and the nutrition status of Chinese children, the Nutrition Improvement Project on Children in Poor Areas of China (NIPCPAC) was launched by the Chinese government in 2012, and YYB was applied in this project to improve the body growth and nutritional status of the 6–23-month-old IYC [9]. In 2012, the baseline study of NIPCPAC was conducted in six counties in Shanxi, Yunnan, and Hubei provinces among 6–23-month-old IYC. Since then, the YYB intervention areas gradually expanded and covered 21 provinces of the western and middle regions of China until 2020. The national surveys were conducted in 2015, 2017, and every year after, which revealed the body growth and nutritional status of 6–23-month-old Chinese IYC under YYB intervention.

Until now, the nutritional effects of YYB have been widely proven [12]. However, most of the previous reports focused on the YYB intervention results of specific areas or just reported some inconsistent years of data, which makes it difficult to evaluate the overall effects of YYB in a timely manner. By conducting a nationwide secular trend study, a big data set was accumulated, which could better help to reveal the nationwide effect of YYB intervention among Chinese IYC.

Therefore, the aim of this study was to investigate whether YYB intervention contributed to the improvement of body growth and nutrition status in a much larger population from broader areas in China. In the present study, the anthropometric data collected in the national secular trend study of NIPCPAC from 2015 to 2020 were analyzed and compared with those of the baseline study in 2012, and the correlation analysis between YYB intake amount and body growth was also conducted. To the best of our knowledge, this is the first research that investigated the association of YYB intervention with body growth and development with a nationwide multi-year data set. The results of this study could provide more scientific and objective evidence on the nutritional effects of YYB intervention in Chinese IYC.

## 2. Materials and Methods

### 2.1. Study Design and Population

The baseline study of NIPCPAC was held in Lanping and Heqing in Yunnan, Changyang and Lichuan in Hubei, and Yonghe and Lan County in Shanxi in 2012. The surveyed IYC were 6–23 months old and selected by probability proportionate to size (PPS) sampling method and random isometric sampling method [13]. About 300 IYC were monitored in each county.

After the baseline study, the YYB product was purchased by the government and delivered to families with 6–23-month-old IYC in the areas the project covered by the three-layer system regularly. IYC started to receive YYB products when they were six months old and up until they were 23 months old. Some nutrition education was also provided along with the YYB product, which included the introduction of YYB and the appropriate intake method. It was recommended to mix the YYB powder with warm water and consume one sachet per day. In the intervention areas, the secular trend study was started in 2015 to evaluate the body growth and nutritional status under YYB intervention. As the major effect of YYB was proven to decrease the anemia rate, the sample size of the annual national survey was estimated based on anemia rate decline from 30% to 24%, 90% power, 95% confidence intervals, a design effect of 3 and 20% nonresponse. A four-layer sampling method was applied in the nationwide cross-sectional surveys, briefly including province stratified sampling method, multistage sampling method, PPS method and systematic random sampling method [14,15]. Individuals with serious acute, chronic and infectious diseases were excluded. This research was approved by the Ethics Committee of the National Institute for Nutrition and Health, Chinese Center for Disease Control and Prevention (No. 2014-001). All caregivers of the IYC gave their informed consent before they participated in the survey.

### 2.2. Data Collection

The surveys were conducted by the doctors in the local maternal and child health hospitals. Each IYC was identified with a unique ID number, and their basic information, such as name, date of birth, gender, YYB consumption situation, etc., was collected by the questionnaire. Furthermore, the body weight and body height of the IYC were measured with a digital physical examination instrument and recorded to the accuracy of 0.05 kg and 0.1 cm, respectively. The instrument was calibrated before the formal test. The clothes and shoes were taken off when measuring. If the IYC wears underclothes, the weight of it should be subtracted. Data for each annual survey was collected from different children aged 6–23 months old in that year, and they were all from the YYB intervention areas. All the doctors and staff for the questionnaire or anthropometric measurement were trained and passed a unified assessment.

### 2.3. Data Handling and Statistical Analysis

All the surveyed data was entered into a computer with double-entry verification. The IYC with missing information, such as ID number, gender, age, etc., were excluded from the analysis.

The monitored IYC were divided into three groups according to their age: 6–11 months, 12–17 months, and 18–23 months. In addition, all the IYC from 2015 to 2020 were further grouped according to their average weekly YYB intake amount. The recommended YYB intake was one sachet per day. Based on the average weekly intake amount, the IYC were divided into 1–3 sachets group and 4–7 sachets group.

The Z scores of length for age (HAZ) and weight for age (WAZ) were calculated with the WHO Anthro software (V3.2.2, WHO, Geneva, Switzerland). Stunting was defined as HAZ < −2, and underweight was defined as WAZ < −2 [16].

The results of anthropometric measurements and Z scores were presented as mean ± standard deviation. The mean values were compared by t-test. The malnutrition status was expressed as a percentage and assessed by Chi-square tests. Correlation analysis was conducted between YYB average weekly intake amount and body weight, body height and Z scores. All data were analyzed using SPSS 22.0 (IBM, Armonk, NY, USA), and the significance of the difference was defined when the *p* value was less than 0.05.

## 3. Results

### 3.1. Characteristics of Surveyed IYC

From 2012 to 2020, a total of 217,448 IYC were enrolled in the survey of NIPCPAC. As per the data shown in Table 1, the gender ratios of boys to girls were 1.08:1 in 2012 and 2017, 1.09:1 in 2015, and 1.07:1 in 2018, 2019 and 2020. All the involved IYC were classified into three age groups. The distributions of age groups were 35.8%, 35.1% and 29.1% in 2012, 32.3%, 35.0% and 32.7% in 2015, 34.6%, 33.5% and 31.9% in 2017, 31.5%, 33.5% and 35.0% in 2018, 33.6%, 32.3% and 34.1% in 2019, and 33.3%, 33.3% and 33.4% in 2020. No significant difference was observed in the gender or age distribution among the surveyed IYC for each year.

### 3.2. Growth Status of Surveyed IYC

In the baseline study, the body length and body weight of the 6–23-month-old IYC were 76.0 ± 6.0 cm and 9.59 ± 1.54 kg, respectively (Table 2). For the surveyed population in each year as a whole, in all the surveys from 2015 to 2020, both body length and body weight of the monitored 6–23-month-old IYC showed a significant increase compared to those of the baseline survey (*p* < 0.05, Table 2). This tendency was the same in both genders among the 6–23-month-old IYC for each year (*p* < 0.05, Table 2). Furthermore, in every survey from 2015 to 2020, the body length exhibited a marked increase in the 6–11 months group and 12–17 months group, and the body weight revealed a notable elevation in the 18–23 months group (compared with 2012, *p* < 0.05, Table 2).

Table 3 presents the Z scores of the survey IYC. For the surveyed population in each year as a whole, in comparison with the results of the baseline survey, the HAZ and WAZ scores of the 6–23-month-old IYC exhibited a significant increase in every nationwide survey since 2015 (*p* < 0.05, Table 3). Such an increase was observed in all age groups and genders in HAZ score (compared with 2012, *p* < 0.05, Table 3).

### 3.3. Prevalence of Malnutrition among Surveyed Infants and Young Children

In the YYB intervention areas, the stunting rate among the 6–23-month-old IYC exhibited continuous decline, from 9.7% in 2012 to 5.3% in 2020 (compared with 2012, *p* < 0.05, Table 4). The lowering tendency of stunting prevalence was more pronounced in girls. The stunting rates from 2015 to 2020 were significantly lower than that of the 2012 survey in the 6–23-month-old girl population (*p* < 0.05, Table 4). In addition, the underweight rate fluctuated from 2012 to 2020. The underweight rate of the 6–23-month-old IYC in 2020 was 2.9%, which was 27.5% lower than that of the baseline study (*p* < 0.05, Table 4).

### 3.4. Comparison of the Growth and Development Status of IYC under Different Intake Levels

The YYB was recommended to intake one sachet per day. Compared to the growth and malnutrition status of the IYC who consumed 1–3 sachets YYB per week, the IYC who consumed 4–7 sachets YYB per week showed significantly higher body length, body weight and Z scores (*p* < 0.05, Table 5). The stunting and underweight rates in the 4–7 sachets group were significantly lower than those of the 1–3 sachets group by 11.4% and 15.2%, respectively (*p* < 0.05, Table 5).

### 3.5. Association between YYB Intake and Body Growth and Development

The results of the correlation analysis between YYB average weekly intake amount and body growth and Z scores are shown in Table 6. According to the results, even though the Pearson correlation coefficient was low, the statistical analysis still indicated that a higher intake amount of YYB may significantly contribute to the promotion of body growth and development (*p* < 0.05).

## 4. Discussion

During the past two decades, the nutritional effects of YYB have been proven by numerous studies in different areas from various aspects, such as promoting body growth, lowering anemia rate, decreasing stunting prevalence or improving intelligence development [4,17,18,19,20]. Based on these findings, the implementation of NIPCPAC continuously spread nationwide in China. Until 2020, IYC from poor rural areas of 21 provinces in China could receive YYB products as their complementary feeding. In 2017, the NIPCPAC was included in the National Nutrition Plan (2017–2030) [21], which indicated the significant impact of YYB on the health of Chinese children.

YYB intervention was a nationwide nutrition project, which made it difficult to carry out pre- and post-intervention comparisons or follow-up surveys in a large population. However, a secular trend study has been conducted in the YYB-covered areas since 2015. A big data set was accumulated to reveal the body growth and nutritional status of the IYC under YYB intervention. Therefore, in this study, we analyzed the anthropometric measurement results of the IYC that consumed YYB from 2012 to 2020 and compared the malnutrition prevalence, which could further prove whether YYB intervention was associated with the promotion of body growth and development of Chinese children.

In some of the previous studies, sprinkles may improve the micronutrient status but are not directly related to body growth promotion [22,23]. Different from the usual sprinkles, YYB is a nutrient-dense powder that provides not only vitamins and minerals but also protein. According to the current formulation, one sachet of YYB contained 3 g protein, and the micronutrients in it accounted for at least 50% of the recommended nutrients intake (RNI) or adequate intake (AI) for 6–12-month-old IYC and at least 30% for 1–3-year-old children [24]. Therefore, YYB could contribute to the nutrient demands of IYC and facilitate the promotion of body growth. The growths of body weight and body length are direct and critical indicators to reveal the growth status of IYC. According to the results in Table 2, the 6–23-month-old IYC under YYB intervention showed a significant increase in both body weight and body length when compared with those of the baseline study, which may partially be attributed to the consumption of YYB.

With the measured anthropometric parameters and the basic information of the IYC, the prevalence of malnutrition was evaluated with the WHO standards. The HAZ score was an indicator of stunting. Stunting is one of the health factors that showed life-long consequences for children [25] and requires effective intervention, especially in developing countries [26]. Whether YYB consumption was helpful in lowering the stunting rate remained unclear in previous reports. A meta-analysis in 2015 indicated that YYB intervention showed an insignificant effect on HAZ score [27], while another meta-analysis in 2019 revealed that YYB consumption was related to the reduction of the prevalence of stunting [12]. In previously published papers, the health benefits of YYB were evaluated in different areas with different sample sizes and intervention durations, which may result in inconsistent results and account for the contradiction of the meta-analyses. In this paper, from the analysis of the baseline study and the five rounds of nationwide surveys of NIPCPAC, a reduction in the stunting rate was observed from 2012 to 2020 among the monitored IYC (Table 4), and the YYB weekly intake amount was positively correlated with the body growth and development indicators (Table 6), which indicate that YYB intervention contributed to the improvement of body growth and nutritional status. In addition, the Chinese government promulgated the China’s Children Development Program (2011–2020), and one of the targets was to lower the stunting rate to under 7% by 2020 [28]. The data in Table 4 shows that the stunting rate of the YYB-intervened IYC was 6.7% in 2019 and 5.3% in 2020, which achieves the set target. Moreover, as reported by WHO, UNICEF and the World Bank in 2020, the stunting rate of children under five years old was 4.5% in eastern Asia [29], which was still lower than the NIPCPAC survey result achieved in 2020, and called for continuous efforts to improve Chinese IYC’s growth.

Being underweight is another manifestation of undernutrition and is related to the development of children’s cognitive and intellectual development [30]. The WAZ score was the predictor of being underweight. In this study, compared with the baseline study in 2012, the WAZ score of the 6–23-month-old IYC showed a significant increase since 2015 (Table 3), but the underweight rate exhibited a marked decrease only in 2020 (Table 4). YYB product may contribute to the adequate nutrient intake for IYC and be helpful for the elevation of the WAZ score. However, the elevated WAZ score may still be under −2, the criteria for determining underweight status, thus showing an insignificant effect on the reduction of the underweight prevalence rate. Moreover, early-life nutrition has a prolonged impact on human health. A report from *Lancet* indicated that we should focus on the benefit of long-term investment rather than the short-term change in childhood nutritional status [31]. Therefore, continuous tracking of children’s health in the YYB intervention-covered areas is still needed for future study.

In the progress of achieving the Millennium Development Goals, China has been regarded to have made a huge contribution due to the dramatic decline in children’s malnutrition [32]. With the setup of the Sustainable Development Goals, childhood nutrition has also been proposed to be essential to meet the goals [33]. Therefore, the long-term effort to improve Chinese children’s nutrition is still urgent and necessary. The results of this study proved that the intervention of YYB was associated with the promotion of body growth and the reduction of the stunting prevalence among the 6–23-month-old IYC in poor rural areas. However, there were also some limitations of the present study. As the results showed in Table 6, although the intake amount of YYB positively correlated the body growth and development, the Pearson correlation coefficients were very low, which indicated more confounding factors. The quality of diet was proved to be associated with the prevalence of malnutrition, such as stunting [14,34]. During the implementation of NIPCPAC, the caregivers may get nutrition knowledge from different channels, which leads to better feeding practices. Thus, improved body growth and nutritional status may be achieved through the combined effects of YYB products and good dietary quality. Furthermore, one sachet of YYB was recommended for intake per day, and good compliance with this was important to achieve the optimal effect [35]. As the data shown in Table 5, the IYC who intake more YYB per week exhibited better body growth and a lower undernutrition prevalence rate than those who intake less. Therefore, it was speculated that with the implementation of the NIPCPAC, the caregivers might gradually have a better understanding of YYB intervention, which may result in better compliance and intervention effects. The potential dose–response curve of YYB intervention also needs to be investigated. Except for the amount and the compliance, the YYB intervention duration was also an important factor that calls for more analysis. Therefore, the contribution of YYB and other factors to the improvement of IYC’s nutrition status needs to be further discussed. Moreover, some biochemical parameters in serum, hair or nail may provide more information to reveal the nutritional effect of YYB from various aspects and were under consideration in our further research.

## 5. Conclusions

Early life nutrition is closely related to the body growth and development of IYC. By analyzing the multi-year data of the NIPCPAC, it was found that among the 6–23-month-old IYC in poor rural areas of China, YYB intervention was associated with the promotion of body growth and the decrease of stunting prevalence. In the future, long-term and continuous efforts are still needed to further reveal the health benefits of YYB in depth.

## Figures and Tables

**Table 1:** Number of surveyed infants and young children.

Year	6–11 Months			12–17 Months			18–23 Months			6–23 Months		
	Boy	Girl	Total	Boy	Girl	Total	Boy	Girl	Total	Boy	Girl	Total
2012	336	322	658	329	317	646	289	245	534	954	884	1838
2015	6310	5784	12,094	6826	6293	13,119	6409	5853	12,262	19,545	17,930	37,475
2017	7423	6803	14,226	7117	6693	13,810	6838	6298	13,136	21,378	19,794	41,172
2018	7379	6776	14,155	7688	7330	15,018	8088	7624	15,712	23,155	21,730	44,885
2019	7859	7346	15,205	7543	7061	14,604	7925	7490	15,415	23,327	21,897	45,224
2020	8044	7567	15,611	8044	7562	15,606	8133	7504	15,637	24,221	22,633	46,854

**Table 2:** Body length and body weight of infants and young children.

	2012	2015	2017	2018	2019	2020
**Body Length (cm)**						
6–11 months						
Boy	71.2 ± 3.6	72.2 ± 9.3 (0.056)	72.1 ± 8.3 (0.055)	71.9 ± 4.2 (0.002)	72.1 ± 9.4 (0.092)	72.3 ± 4.4 (<0.001)
Girl	69.5 ± 3.4	71.1 ± 13.9 (0.043)	70.9 ± 13.4 (0.066)	70.7 ± 4.3 (<0.001)	70.7 ± 4.2 (<0.001)	70.9 ± 4.3 (<0.001)
All	70.4 ± 3.6	71.7 ± 11.7 (0.005)	71.5 ± 11.0 (0.009)	71.3 ± 4.3 (<0.001)	71.4 ± 7.4 (<0.001)	71.6 ± 4.4 (<0.001)
12–17 months						
Boy	77.6 ± 3.4	78.6 ± 14.7 (0.229)	78.1 ± 10.1 (0.358)	78.1 ± 9.1 (0.321)	78.1 ± 4.1 (0.010)	78.4 ± 7.9 (0.076)
Girl	75.7 ± 3.5	77.1 ± 10.3 (0.014)	76.8 ± 9.3 (0.025)	76.9 ± 9.1 (0.014)	76.9 ± 4.1 (<0.001)	77.2 ± 4.0 (<0.001)
All	76.7 ± 3.5	77.9 ± 12.8 (0.016)	77.5 ± 9.8 (0.028)	77.5 ± 9.1 (0.015)	77.5 ± 4.1 (<0.001)	77.8 ± 6.3 (<0.001)
18–23 months						
Boy	82.8 ± 3.6	83.5 ± 10.9 (0.314)	83.6 ± 16.4 (0.422)	83.5 ± 4.5 (0.010)	83.6 ± 4.1 (0.001)	84.1 ± 4.2 (<0.001)
Girl	81.5 ± 3.7	82.6 ± 16.8 (0.305)	82.6 ± 17.1 (0.339)	82.6 ± 4.5 (<0.001)	82.7 ± 9.8 (0.071)	83.2 ± 13.6 (0.058)
All	82.2 ± 3.7	83.1 ± 14.1 (0.167)	83.1 ± 16.7 (0.226)	83.1 ± 4.5 (<0.001)	83.2 ± 7.4 (0.004)	83.7 ± 9.9 (<0.001)
6–23 months						
Boy	76.9 ± 5.9	78.1 ± 12.8 (0.004)	77.8 ± 12.9 (0.044)	78.0 ± 7.9 (<0.001)	78.0 ± 8.0 (<0.001)	78.3 ± 7.5 (<0.001)
Girl	75.1 ± 5.9	77.0 ± 14.6 (<0.001)	76.6 ± 14.3 (0.001)	77.0 ± 8.0 (<0.001)	76.8 ± 8.3 (<0.001)	77.1 ± 9.9 (<0.001)
Total	76.0 ± 6.0	77.6 ± 13.7 (<0.001)	77.2 ± 13.6 (<0.001)	77.5 ± 8.0 (<0.001)	77.4 ± 8.1 (<0.001)	77.7 ± 8.7 (<0.001)
**Body weight (kg)**						
6–11 months						
Boy	8.86 ± 1.22	9.07 ± 2.02 (0.070)	8.97 ± 1.82 (0.316)	9.05 ± 2.71 (0.202)	8.99 ± 2.48 (0.374)	9.07 ± 2.05 (0.063)
Girl	8.21 ± 1.11	8.64 ± 3.61 (0.033)	8.47 ± 2.40 (0.052)	8.54 ± 2.94 (0.048)	8.48 ± 2.88 (0.099)	8.59 ± 2.98 (0.024)
All	8.54 ± 1.21	8.86 ± 2.90 (<0.001)	8.73 ± 2.13 (0.027)	8.81 ± 2.83 (0.018)	8.74 ± 2.70 (0.064)	8.84 ± 2.56 (0.003)
12–17 months						
Boy	9.96 ± 1.15	10.25 ± 1.96 (<0.001)	10.14 ± 2.07 (0.120)	10.23 ± 2.91 (0.098)	10.11 ± 2.09 (0.206)	10.25 ± 2.25 (0.018)
Girl	9.31 ± 1.17	9.67 ± 2.06 (0.002)	9.50 ± 1.88 (0.069)	9.66 ± 2.56 (0.015)	9.54 ± 1.47 (0.005)	9.81 ± 10.37 (0.385)
All	9.64 ± 1.21	9.97 ± 2.03 (<0.001)	9.83 ± 2.01 (<0.001)	9.95 ± 2.76 (0.005)	9.83 ± 1.84 (0.008)	10.04 ± 7.40 (0.168)
18–23 months						
Boy	11.08 ± 1.30	11.30 ± 1.85 (0.007)	11.21 ± 2.14 (0.276)	11.33 ± 2.52 (0.094)	11.27 ± 1.73 (0.063)	11.44 ± 1.90 (<0.001)
Girl	10.51 ± 1.26	10.72 ± 2.06 (0.115)	10.65 ± 1.52 (0.152)	10.80 ± 1.98 (0.023)	10.73 ± 2.25 (0.116)	10.87 ± 1.81 (0.002)
All	10.82 ± 1.31	11.02 ± 1.98 (<0.001)	10.94 ± 1.89 (0.031)	11.07 ± 2.29 (0.011)	11.01 ± 2.02 (0.028)	11.17 ± 1.88 (<0.001)
6–23 months						
Boy	9.91 ± 1.51	10.21 ± 2.15 (<0.001)	10.08 ± 2.21 (0.002)	10.24 ± 2.87 (<0.001)	10.12 ± 2.32 (<0.001)	10.26 ± 2.29 (<0.001)
Girl	9.24 ± 1.49	9.68 ± 2.79 (<0.001)	9.51 ± 2.17 (<0.001)	9.71 ± 2.67 (<0.001)	9.59 ± 2.47 (<0.001)	9.75 ± 6.39 (0.017)
Total	9.59 ± 1.54	9.96 ± 2.49 (<0.001)	9.80 ± 2.21 (<0.001)	9.98 ± 2.79 (<0.001)	9.87 ± 2.41 (<0.001)	10.02 ± 4.75 (<0.001)

Data were expressed as mean ± SD. The mean values were compared with the data in 2012 by *t*-test, and the *p* values were presented in brackets.

**Table 3:** The Z scores of infants and young children.

	2012	2015	2017	2018	2019	2020
**HAZ**						
6–11 months						
Boy	−0.42 ± 1.21	−0.13 ± 1.38 (<0.001)	−0.16 ± 1.34 (<0.001)	−0.13 ± 1.36 (<0.001)	−0.10 ± 1.30 (<0.001)	−0.05 ± 1.26 (<0.001)
Girl	−0.25 ± 1.06	0.08 ± 1.31 (<0.001)	0.01 ± 1.27 (<0.001)	0.07 ± 1.29 (<0.001)	0.07 ± 1.23 (<0.001)	0.11 ± 1.21 (<0.001)
All	−0.33 ± 1.14	−0.03 ± 1.35 (<0.001)	−0.08 ± 1.31 (<0.001)	−0.04 ± 1.33 (<0.001)	−0.02 ± 1.27 (<0.001)	0.03 ± 1.24 (<0.001)
12–17 months						
Boy	−0.58 ± 1.15	−0.28 ± 1.38 (<0.001)	−0.36 ± 1.32 (<0.001)	−0.38 ± 1.31 (0.002)	−0.34 ± 1.27 (<0.001)	−0.24 ± 1.26 (<0.001)
Girl	−0.62 ± 1.07	−0.14 ± 1.31 (<0.001)	−0.20 ± 1.22 (<0.001)	−0.17 ± 1.24 (<0.001)	−0.16 ± 1.22 (<0.001)	−0.06 ± 1.18 (<0.001)
All	−0.60 ± 1.11	−0.21 ± 1.35 (<0.001)	−0.28 ± 1.28 (<0.001)	−0.28 ± 1.28 (<0.001)	−0.25 ± 1.25 (<0.001)	−0.15 ± 1.23 (<0.001)
18–23 months						
Boy	−0.80 ± 1.13	−0.52 ± 1.34 (<0.001)	−0.55 ± 1.30 (<0.001)	−0.54 ± 1.25 (<0.001)	−0.48 ± 1.24 (<0.001)	−0.31 ± 1.21 (<0.001)
Girl	−0.61 ± 1.11	−0.39 ± 1.32 (0.002)	−0.41 ± 1.24 (0.009)	−0.35 ± 1.20 (<0.001)	−0.34 ± 1.16 (<0.001)	−0.20 ± 1.16 (<0.001)
All	−0.71 ± 1.12	−0.46 ± 1.34 (<0.001)	−0.48 ± 1.27 (<0.001)	−0.44 ± 1.23 (<0.001)	−0.41 ± 1.21 (<0.001)	−0.26 ± 1.19 (<0.001)
6–23 months						
Boy	−0.59 ± 1.17	−0.31 ± 1.38 (<0.001)	−0.35 ± 1.33 (<0.001)	−0.35 ± 1.32 (<0.001)	−0.31 ± 1.28 (<0.001)	−0.20 ± 1.25 (<0.001)
Girl	−0.48 ± 1.09	−0.15 ± 1.33 (<0.001)	−0.19 ± 1.26 (<0.001)	−0.16 ± 1.26 (<0.001)	−0.14 ± 1.21 (<0.001)	−0.05 ± 1.19 (<0.001)
Total	−0.54 ± 1.14	−0.23 ± 1.36 (<0.001)	−0.27 ± 1.30 (<0.001)	−0.26 ± 1.29 (<0.001)	−0.23 ± 1.25 (<0.001)	−0.13 ± 1.22 (<0.001)
**WAZ**						
6–11 months						
Boy	−0.14 ± 1.14	0.01 ± 1.27 (0.043)	−0.09 ± 1.23 (0.503)	−0.03 ± 1.19 (0.122)	−0.06 ± 1.18 (0.225)	0.02 ± 1.16 (0.014)
Girl	−0.06 ± 1.01	0.11 ± 1.17 (0.004)	0.04 ± 1.14 (0.072)	0.08 ± 1.16 (0.025)	0.04 ± 1.07 (0.101)	0.13 ± 1.06 (<0.001)
All	−0.10 ± 1.08	0.06 ± 1.23 (<0.001)	−0.03 ± 1.19 (0.121)	0.02 ± 1.16 (0.009)	−0.01 ± 1.13 (0.048)	0.08 ± 1.11 (<0.001)
12–17 months						
Boy	−0.36 ± 1.01	−0.15 ± 1.20 (<0.001)	−0.24 ± 1.16 (0.036)	−0.20 ± 1.12 (0.011)	−0.25 ± 1.11 (0.053)	−0.13 ± 1.07 (<0.001)
Girl	−0.31 ± 0.97	−0.06 ± 1.09 (<0.001)	−0.16 ± 1.07 (0.009)	−0.08 ± 1.04 (<0.001)	−0.11 ± 1.02 (<0.001)	0.01 ± 1.00 (<0.001)
All	−0.33 ± 0.99	−0.11 ± 1.15 (<0.001)	−0.20 ± 1.12 (<0.001)	−0.14 ± 1.09 (<0.001)	−0.18 ± 1.07 (<0.001)	−0.06 ± 1.04 (<0.001)
18–23 months						
Boy	−0.44 ± 1.01	−0.27 ± 1.14 (0.007)	−0.35 ± 1.11 (0.174)	−0.29 ± 1.07 (0.024)	−0.29 ± 1.06 (0.019)	−0.16 ± 1.02 (<0.001)
Girl	−0.30 ± 0.96	−0.20 ± 1.07 (0.168)	−0.25 ± 1.06 (0.469)	−0.15 ± 1.00 (0.024)	−0.20 ± 0.98 (0.130)	−0.07 ± 0.97 (<0.001)
All	−0.37 ± 0.99	−0.24 ± 1.11 (0.002)	−0.30 ± 1.09 (0.123)	−0.22 ± 1.04 (0.001)	−0.25 ± 1.02 (0.005)	−0.12 ± 1.00 (<0.001)
6–23 months						
Boy	−0.31 ± 1.06	−0.14 ± 1.21 (<0.001)	−0.22 ± 1.17 (0.020)	−0.18 ± 1.13 (<0.001)	−0.20 ± 1.12 (0.002)	−0.09 ± 1.09 (<0.001)
Girl	−0.22 ± 0.99	−0.05 ± 1.11 (<0.001)	−0.12 ± 1.10 (0.005)	−0.05 ± 1.06 (<0.001)	−0.09 ± 1.03 (<0.001)	0.02 ± 1.02 (<0.001)
Total	−0.26 ± 1.03	−0.10 ± 1.17 (<0.001)	−0.17 ± 1.14 (<0.001)	−0.12 ± 1.10 (<0.001)	−0.15 ± 1.08 (<0.001)	−0.03 ± 1.05 (<0.001)

Data were expressed as mean ± SD. The mean values were compared with the data in 2012 by *t*-test, and the *p* values were presented in brackets. HAZ: Z score of length for age. WAZ: Z score of weight for age.

**Table 4:** Malnutrition status of infants and young children.

	2012	2015	2017	2018	2019	2020
**Stunting**						
6–11 months						
Boy	8.4	7.7 (0.655)	7.2 (0.416)	7.5 (0.569)	5.9 (0.059)	5.2 (0.010)
Girl	2.5	4.1 (0.148)	4.6 (0.070)	4.2 (0.137)	3.5 (0.326)	3.0 (0.570)
All	5.5	6.0 (0.595)	6.0 (0.609)	5.9 (0.645)	4.7 (0.374)	4.1 (0.091)
12–17 months						
Boy	10.3	9.6 (0.654)	9.3 (0.545)	9.5 (0.611)	8.3 (0.199)	7.4 (0.049)
Girl	11.4	6.5 (<0.001)	6.1 (<0.001)	5.5 (<0.001)	5.5 (<0.001)	4.0 (<0.001)
All	10.8	8.1 (0.013)	7.8 (0.005)	7.5 (0.002)	7.0 (<0.001)	5.8 (<0.001)
18–23 months						
Boy	14.2	12.2 (0.307)	11.5 (0.152)	11.1 (0.094)	10.0 (0.019)	7.0 (<0.001)
Girl	13.1	10.0 (0.117)	8.4 (0.011)	7.3 (<0.001)	6.8 (<0.001)	5.1 (<0.001)
All	13.7	11.1 (0.068)	10.0 (0.006)	9.2 (<0.001)	8.4 (<0.001)	6.1 (0.001)
6–23 months						
Boy	10.8	9.8 (0.324)	9.3 (0.109)	9.4 (0.148)	8.1 (0.002)	6.5 (<0.001)
Girl	8.6	6.9 (0.049)	6.3 (0.007)	5.7 (<0.001)	5.3 (<0.001)	4.1 (<0.001)
Total	9.7	8.4 (0.046)	7.9 (0.003)	7.6 (<0.001)	6.7 (<0.001)	5.3 (<0.001)
**Underweight**						
6–11 months						
Boy	4.5	5.0 (0.648)	5.6 (0.400)	4.1 (0.768)	4.7 (0.854)	3.4 (0.315)
Girl	2.8	3.3 (0.629)	3.4 (0.561)	3.0 (0.839)	3.2 (0.720)	2.1 (0.435)
All	3.7	4.2 (0.495)	4.5 (0.295)	3.6 (0.936)	3.9 (0.702)	2.8 (0.209)
12–17 months						
Boy	3.6	5.4 (0.174)	6.0 (0.074)	5.0 (0.256)	5.7 (0.116)	3.7 (0.998)
Girl	4.1	3.6 (0.624)	4.4 (0.804)	3.0 (0.277)	3.4 (0.484)	2.6 (0.092)
All	3.9	4.5 (0.445)	5.2 (0.126)	4.1 (0.815)	4.6 (0.406)	3.1 (0.286)
18–23 months						
Boy	5.2	5.9 (0.595)_	6.3 (0.436)	4.9 (0.838)	4.8 (0.755)	3.2 (0.056)
Girl	4.1	5.0 (0.537)	4.6 (0.725)	3.4 (0.557)	3.8 (0.830)	2.5 (0.117)
All	4.7	5.5 (0.432)	5.5 (0.427)	4.2 (0.569)	4.3 (0.683)	2.8(0.013)
6–23 months						
Boy	4.4	5.4 (0.165)	6.0 (0.047)	4.7 (0.660)	5.0 (0.375)	3.4 (0.103)
Girl	3.6	3.9 (0.640)	4.1 (0.479)	3.1 (0.427)	3.4 (0.786)	2.4 (0.021)
Total	4.0	4.7 (0.169)	5.1 (0.047)	4.0 (0.872)	4.3 (0.610)	2.9 (0.006)

The malnutrition rates were compared with the data in 2012 by Chi-square test, and the *p* values were presented in brackets.

**Table 5:** Comparison of the growth and development status of infants and young children under different YYB intake levels.

Average Intake/Week (Sachets)	Body Length (cm)	Body Weight (kg)	HAZ	WAZ	Stunting (%)	Underweight (%)
1–3	77.1 ± 10.4	9.84 ± 5.96	−0.29 ± 1.27	−0.17 ± 1.12	7.9	4.6
4–7	78.6 ± 10.5 *	10.14 ± 2.33 *	−0.23 ± 1.26 *	−0.12 ± 1.08 *	7.0 *	3.9 *
*p* value	<0.001	<0.001	<0.001	<0.001	<0.001	<0.001

Data of 4–7 sachets/week group were compared with the data of 1–3 sachets/week and marked with * when significantly different (*p* < 0.05).

**Table 6:** Correlation coefficient between YYB intake amount and body growth indicators.

		Body Length	Body Weight	HAZ	WAZ
Average weekly intake amount	Pearson correlation coefficient	0.074	0.046	0.015	0.016
	*p* value	<0.001	<0.001	<0.001	<0.001

## Data Availability

Data sharing is not applicable to this article.
